# Long non-coding RNA *UCA1* is a predictive biomarker of cancer

**DOI:** 10.18632/oncotarget.10142

**Published:** 2016-06-17

**Authors:** Han-han Hong, Li-kun Hou, Xin Pan, Chun-yan Wu, Hai Huang, Bing Li, Wei Nie

**Affiliations:** ^1^ Department of Respiratory Medicine, Shanghai Changzheng Hospital, Second Military Medical University, Shanghai, China; ^2^ Department of Pathology, Shanghai Pulmonary Hospital, Tongji University School of Medicine, Shanghai, China; ^3^ Department of Medical Section, Zhenjiang Emergency Medical Center, Zhenjiang, Jiangsu, China

**Keywords:** cancer, UCA1, biomarker

## Abstract

Human urothelial carcinoma associated 1 (*UCA1*) is a long noncoding RNA that is putatively oncogenic in solid tumors. This meta-analysis investigated an association between *UCA1* levels and survival times of cancer patients. The primary endpoints were overall survival (OS) and progression-free survival (PFS). A comprehensive, computerized literature search was conducted of the databases PubMed, EMBASE, Chinese National Knowledge Infrastructure (CNKI), and Wanfang. The strength of association between *UCA1* and cancer prognosis was assessed by computing the hazard ratio (HR) with its corresponding 95% confidence interval (CI). Twelve studies comprising 954 cancer patients met the criteria for this meta-analysis. Overall, a significant negative association was found between *UCA1* levels and OS time (HR1.81, 95% CI1.52−2.17), including the following cancers analyzed independently: colorectal (HR2.61, 95% CI1.56−4.37), non-small cell lung (HR1.49, 95% CI1.16−1.90), gastric (HR2.19, 95% CI1.36−3.51), and ovarian (HR1.89, 95% CI1.14−3.12). There was also a significant negative association between UCA1 levels and PFS time (HR2.59, 95% CI1.61−4.16). In conclusion, this meta-analysis indicated that higher levels of *UCA1* correlate with shorter PFS and OS times in cancers.

## INTRODUCTION

Cancer is a major public health problem worldwide, with overall death rates that rose during most of the 20 th century. In the United States, cancer is the second leading cause of death [1]. In China, cancers are the leading cause of death, despite the development of effective drugs and supportive care [2].

Long noncoding RNAs (lncRNAs) are non-protein-coding molecules, longer than 200 nucleotides [3]. Many studies have reported that lncRNAs are deregulated in cancers, suggesting that the aberrant expression of lncRNAs is associated with tumorigenesis, metastasis, and prognosis in cancer.

Human *UCA1* (urothelial carcinoma associated 1) is a lncRNA that was first identified in human bladder carcinoma [4], and whose oncogenic effect may be related to glucose metabolism [5]. Recently, some studies have reported the relevance of *UCA1* in cancer prognosis and the acquired resistance to drugs [6–17]. For example, patients with advanced non-small cell lung cancer (NSCLC) that harbor mutations that activate epidermal growth factor receptor (*EGFR*) can be treated with *EGFR*-tyrosine kinase inhibitors (TKIs) such as gefitinib. However resistance to this treatment is often acquired, most commonly via a secondary *T790M* mutation. Cheng et al. found that the lncRNA *UCA1* was upregulated in resistant cells, and that overexpression of *UCA1 was associated with shorter* progression-free survival (PFS) in non-resistant cells [8]. Furthermore, *UCA1* knockdown restored sensitivity to gefitinib in acquired-resistant NSCLC cells without the *T790M* mutation, and inhibited the activation of the AKT/mTOR pathway and epithelial-mesenchymal transition.

No meta-analysis was been conducted to assess the association between *UCA1* and the survival of patients with cancers. Therefore, this meta-analysis investigated an association between *UCA1* and the survival of cancer patients. Overall survival (OS) and PFS were the primary endpoints.

## RESULTS

### Study characteristics

**Figure 1:**
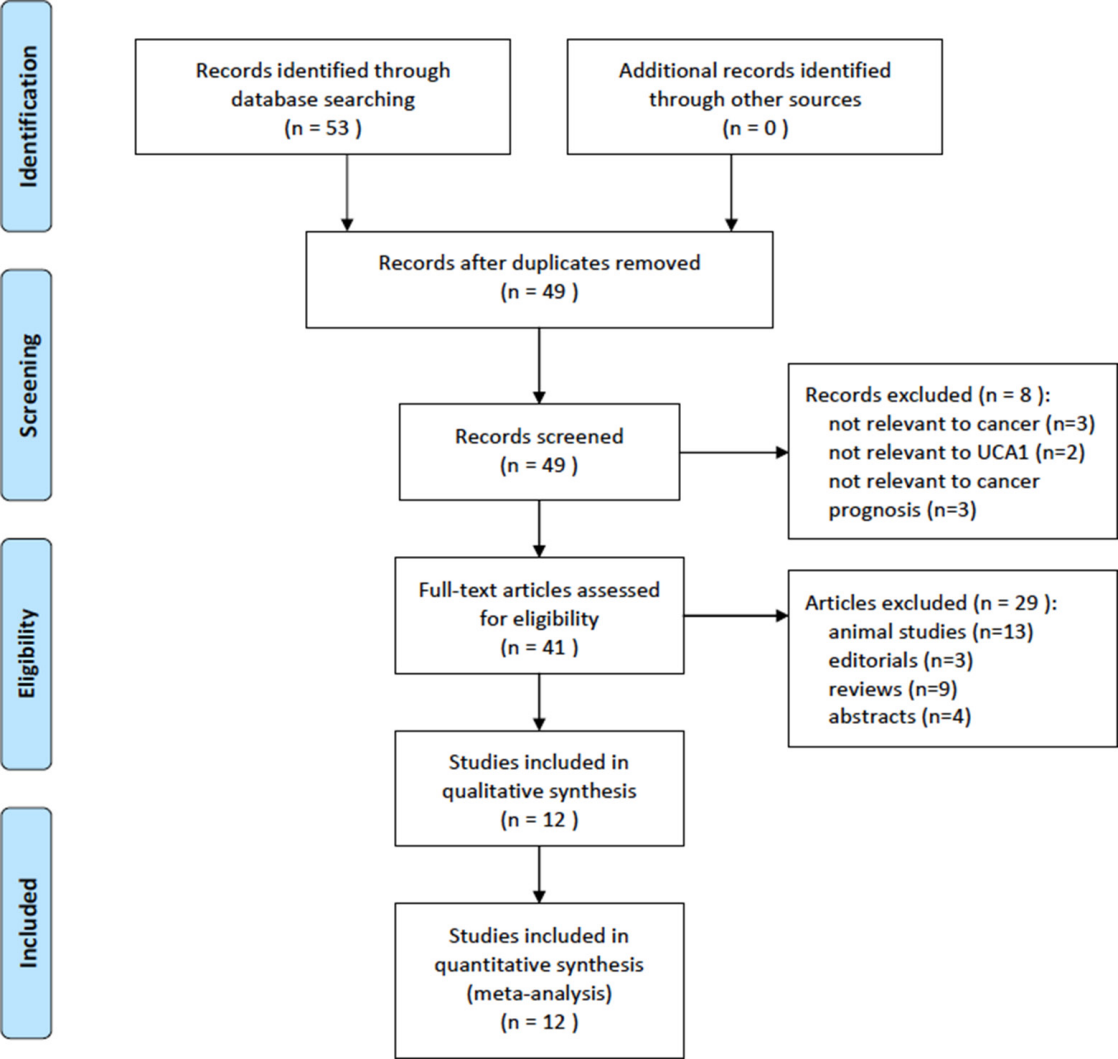
Flow of study selection

The initial search of the databases produced 53 studies (Figure 1). After excluding duplicate articles, 49 potentially eligible studies were selected. After a detailed evaluation, 12 studies were selected for the final meta-analysis with a total of 954 cancer patients (Table 1). Of the 12 studies, 2, 3, 2, and 2 concerned colorectal cancer, NSCLC, ovarian cancer, and gastric cancer, respectively, and there was one study each regarding esophageal squamous cell carcinoma and hepatocellular carcinoma.

**Table 1:** Characteristics of the included studies^a^

First author	Year	n^b^	Age^c^	Men, %	Reference	FU, mo	Cancer	Outcome	Co-variants	NOS
Cheng	2015	94	NA*	46.4	GAPDH	24	NSCLC	PFS	Age	7
Gao	2015	20	NA	NA	GAPDH	NA	GC	OS	Lymph node; clinical stage	8
Han	2014	80	55	49	GAPDH	42.6	CRC	OS	NA	8
Kamel	2015	82	57	68.3	GAPDH	NA	HCC	PFS	Barcelona clinic liver Cancer stage;Child score; mean tumor size	8
Li	2014	90	60	55.6	GAPDH	43	ESCC	OS	Differentiation grade; lymph node; clinical stage	7
Ni	2015	54	NA	72.2	GAPDH	NA	CRC	OS	Lymphatic invasion; lymph node; distant metastasis; clinical stage	8
Nie	2015	112	63.2	59.8	GAPDH	45	NSCLC	OS	Lymph node; clinical stage	8
Tao	2015	80	65.1	60	RUN6	NA	CRC	OS	Lymph node; clinical stage	7
Wang	2015	60	NA	61.7	GAPDH	NA	NSCLC	OS	Lymph node; clinical stage	7
Yang	2016	53	NA	0	GAPDH	NA	Ovary	OS	Lymph node	8
Zhang	2016	117	33	0	RUN6	22	Ovary	OS	Chemotherapy response; lymph node; clinical stage	8
Zheng	2015	112	NA	57.1	GAPDH	NA	GC	OS, PFS	Tumor size; invasion depth; lymphatic metastasis; invade adjacent organs; clinical stage	8

a This study characterized patients as > 65 or < 65; technique used to quantify *UCA1* was real-time PCR in all studies;

b sample size;

c median age, y.

CRC, colorectal cancer; ESCC, esophageal squamous cell carcinoma; FU, follow-up; GAPDH, glyceraldehyde-3-phosphate dehydrogenase; GC, gastric cancer; HCC, hepatocellular carcinoma; NA, not available.

Not all studies examined both OS and PFS, because most of the studies were retrospective cohort studies; 10 studies investigated the association between *UCA1* and OS, while 3 studies assessed the association between *UCA1* and PFS.

### Results of the meta-analysis

**Figure 2:**
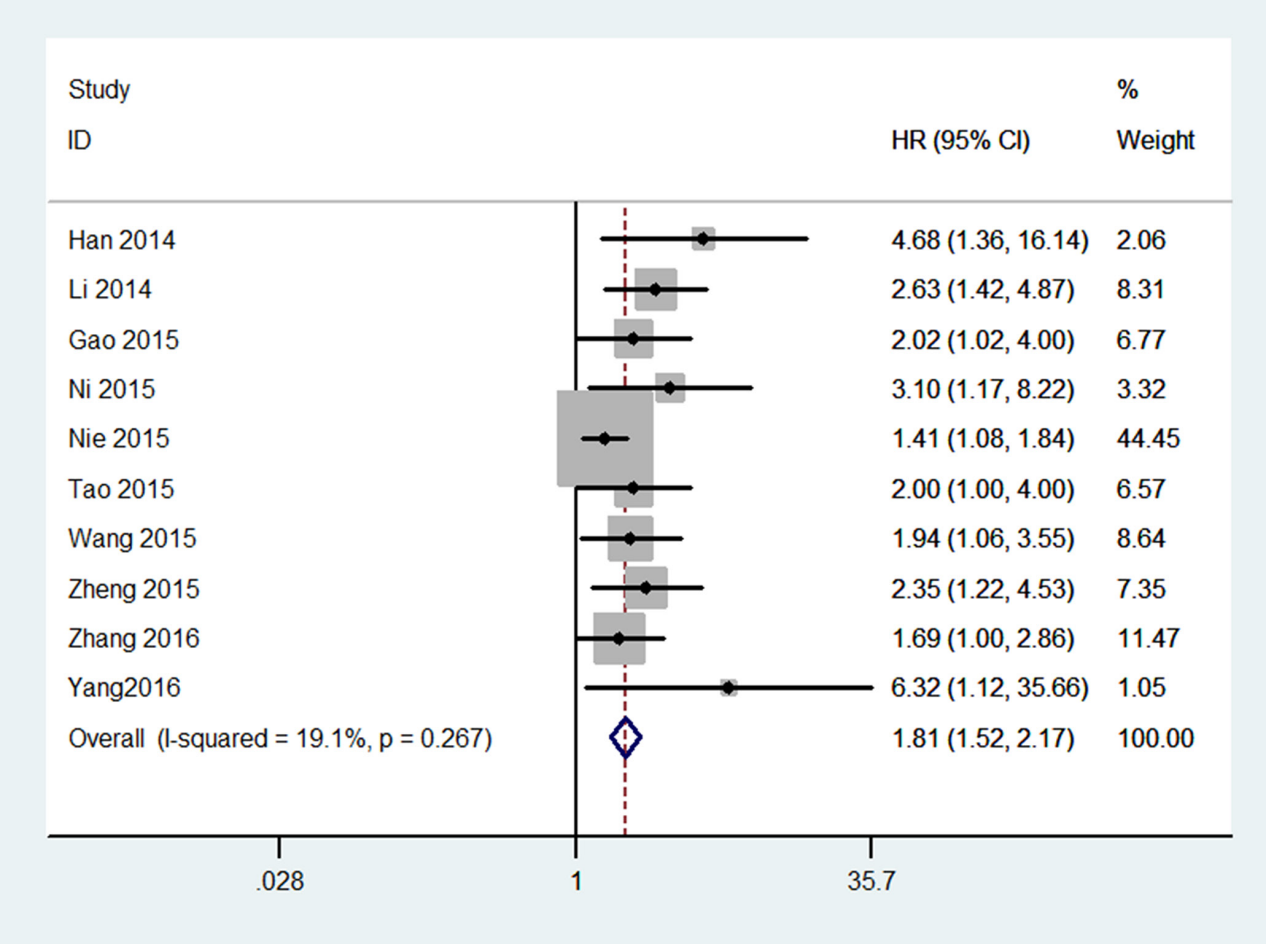
Meta-analysis for the association between *UCA1* and overall survival of cancer

The association between the expression of *UCA1* and OS was investigated in 10 studies (Figure 2). We found a statistically significant negative association between levels of *UCA1* and OS (HR = 1.81, 95% CI = 1.52–2.17). In a subgroup analysis of cancer sites, significant negative associations were found between levels of *UCA1* and OS in the following cancers: colorectal (HR2.61, 95% CI1.56–4.37), NSCLC (HR1.49, 95% CI1.16-1.90), gastric cancer (HR2.19, 95% CI1.36–3.51), and ovarian cancer (HR1.89, 95% CI1.14–3.12). When the studies that adjusted for lymph node and clinical stage were included, shorter OS was also observed (HR1.71, 95% CI = 1.42–2.07). We did not perform subgroup analyses for esophageal squamous cell carcinoma or hepatocellular carcinoma, because no more than one study each investigated these associations between *UCA1* and OS.

**Figure 3:**
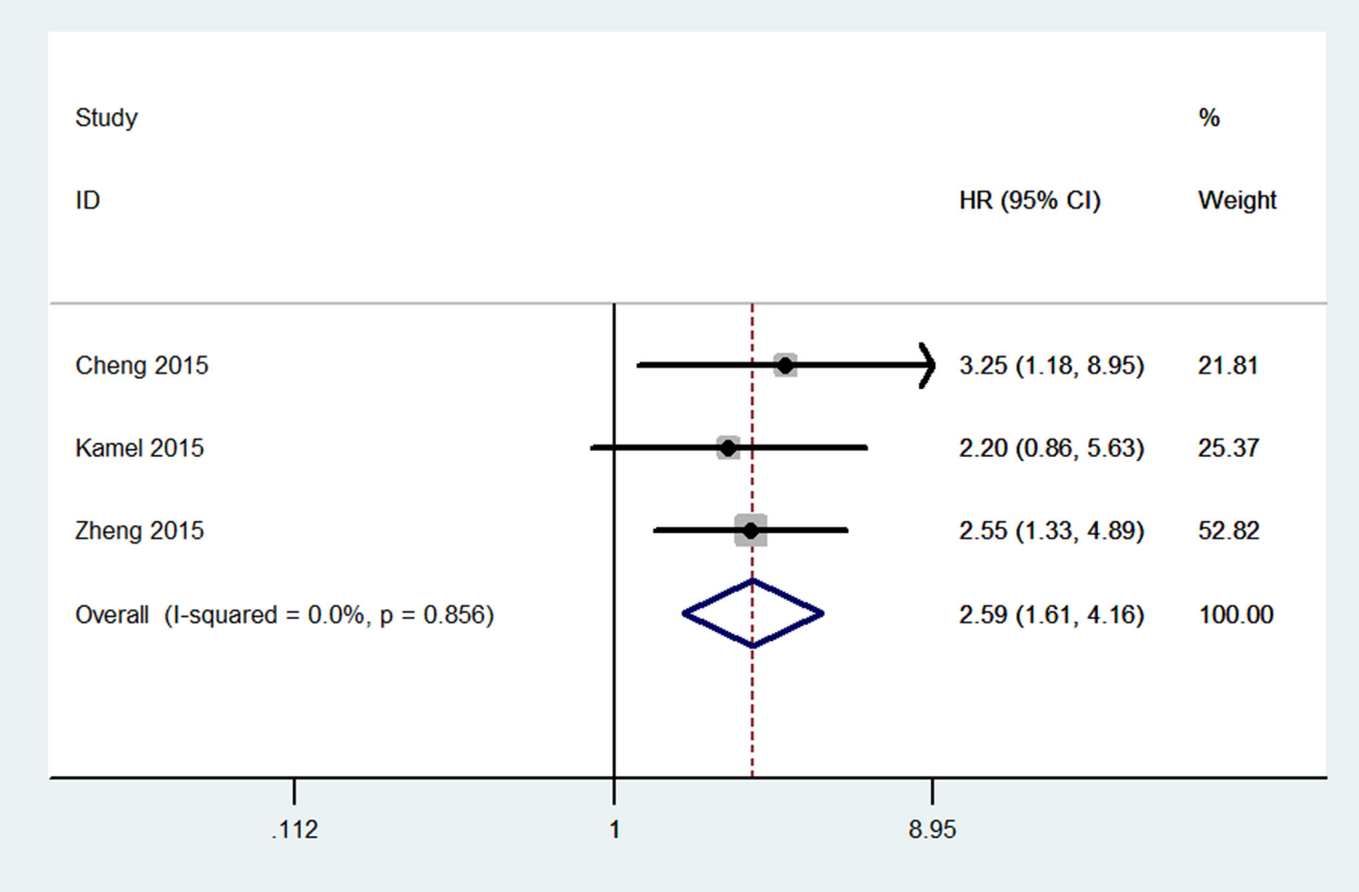
Meta-analysis for the association between *UCA1* and progression-free survival of cancer

The association between *UCA1* and PFS was investigated in 3 studies (Figure 3). There was a significant negative association between *UCA1* levels and PFS (HR2.59, 95% CI1.61–4.16; Figure 3). All the results are listed in the Table 2.

**Table 2:** Results of this meta-analysis

		HR (95% CI)	*P*	I^2^ (%)	*P*
Overall survival		1.81 (1.52–2.17)	< 0.00001	19	0.27
Site of cancer	CRC	2.62 (1.56–4.37)	0.0002	0	0.46
	NSCLC	1.49 (1.16–1.90)	0.001	0	0.34
	GC	2.19 (1.36–3.51)	0.001	0	0.75
	Ovary	1.89 (1.14–3.12)	0.01	51	0.15
Adjusted lymph node and clinical stage		1.71 (1.42–2.07)	< 0.00001	0	0.43
Progression-free survival		2.59 (1.61–4.16)	< 0.00001	0	0.86

CRC, colorectal cancer; NSCLC, non-small cell lung cancer; GC, gastric cancer.

## DISCUSSION

This is the first meta-analysis to evaluate the association between *UCA1* levels and cancer prognosis. We found that increased levels of *UCA1* were significantly associated with shorter OS and PFS times in cancer patients. In the subgroup analyses, *UCA1* levels were significantly and negatively associated with OS times in colorectal cancer, NSCLC, ovarian cancer, and gastric cancer.

*UCA1* putatively influences the proliferation, apoptosis, and cell cycle progression of colorectal cancer cells [6]. Ni et al. [11]also found that knockdown of *UCA1* was associated with suppressed cell proliferation and metastasis in colorectal cancer cells. Nie et al. [12] suggested that silencing of *UCA1* impaired the proliferation and colony formation of NSCLC cells. Wang and coworkers [13] found that *UCA1* levels were associated with histological grade and lymph node metastasis in NSCLC. In addition, a clinicopathologic analysis revealed that *UCA1* levels correlated with worse differentiation, greater tumor size and invasion depth, and TNM stage in gastric cancer [14]. Thus, these data might explain why high levels of *UCA1* were significantly associated with shorter OS and PFS in cancer patients in this meta-analysis.

The clinical implications of *UCA1* in various cancers have not been studied well. Wang et al. [4] showed that a *UCA1* assay was highly specific (91.8%, 78 of 85) and very sensitive (80.9%, 76 of 94) in the diagnosis of bladder cancer. However, Milowich et al. [18] indicated that the efficiency of the *UCA1* test for detecting primary and recurring bladder cancer was low. Future studies should focus on the clinical utility of *UCA1*-based cancer diagnosis in clinical trials. In addition, *UCA1* has been implicated in the acquired resistance to EGFR-TKIs in EGFR-mutant NSCLC that did not include a *T790M* mutation [8]. Thus, the expression of *UCA1* should be evaluated before patients receive EGFR-TKIs.

Some limitations of this meta-analysis should be pointed out. Firstly, the number of included studies in our meta-analysis was moderate. Secondly, most of the studies were conducted with Chinese sample populations and, therefore, our results may be applicable only to this ethnic group. Thirdly, not all of the studies reported the cutoff values of *UCA1*. Finally, many factors, such as gender and chemotherapy, may also affect OS and PFS. Thus, the results of this meta-analysis should be confirmed in future studies.

In conclusion, the results of this meta-analysis suggest that *UCA1* may be a risk factor for shorter OS and PFS in cancers. Well-designed studies with large sample sizes are needed to confirm further the association between *UCA1* and clinical outcomes of cancers in various ethnic populations.

## MATERIALS AND METHODS

### Publication search

We searched the databases PubMed, EMBASE, Chinese National Knowledge Infrastructure (CNKI) and Wanfang, to 14 March 2016, for relevant articles. The search terms used were “*UCA1*” and “cancer or carcinoma or tumor”. Reference lists of relevant articles were also reviewed to identify potential eligible studies.

### Inclusion and exclusion criteria

For inclusion in this meta-analysis, the studies met the following criteria: cohort design; investigated the association between *UCA1* and cancer prognosis (OS or PFS); and sufficient original data for calculating a hazard ratio (HR) with its 95% confidence interval (CI). A study was excluded if it was not relevant to cancer, *UCA1*, or cancer prognosis; involved animals; or was an editorial, review, or abstract. If more than one study used the same patient cases, the one with the most comprehensive population was included. Differences in opinion among the authors were solved by discussion.

### Data extraction and quality assessment

Two investigators extracted and reviewed the data independently. The following data were extracted: the first author's name, year of publication, patient ages and genders, duration of follow-up, sample size, site of cancer, PFS, OS, and co-variants. Since all included studies were cohort studies, the Newcastle-Ottawa Scale (NOS) was used to evaluate the methodological quality [19].

### Statistical analysis

The strength of association between *UCA1* and cancer prognosis (PFS and OS) was assessed by computing the HR with its corresponding 95% CI. OS was defined as the time between diagnosis and death. PFS was defined as the time between diagnosis and progression. The heterogeneity among eligible studies was checked by using the chi-squared based *Q*-statistic test. The random-effects model or fixed-effects model was used to analyze the pooled HRs. If the number of included studies in an analysis was more than 10, Egger's linear regression test and Begg's funnel-plot analysis were used to weigh the potential publication bias. All the *P*-values were determined by a 2-sided test. All statistical analyses were conducted using STATA software (version 12.0; Stata, College Station, Texas).
